# Modulatory effects of SES and multilinguistic experience on cognitive development: a longitudinal data analysis of multilingual and monolingual adolescents from the SCAMP cohort

**DOI:** 10.1080/13670050.2022.2064191

**Published:** 2022-04-20

**Authors:** Roberto Filippi, Andrea Ceccolini, Elizabeth Booth, Chen Shen, Michael S.C. Thomas, Mireille B. Toledano, Iroise Dumontheil

**Affiliations:** a UCL – Institute of Education, University College London, London, UK; b Centre for Brain and Cognitive Development, Department of Psychological Sciences, Birkbeck College, London, UK; c Multilanguage and Cognition Lab, UCL – Institute of Education, London, UK; d MRC Centre for Environment and Health, Department of Epidemiology and Biostatistics, School of Public Health, Imperial College, London, UK; e National Institute for Health Research Health Protection Research Unit in Chemical and Radiation Threats and Hazards, Imperial College London, London, UK; f Mohn Centre for Children’s Health and Wellbeing, School of Public Health, Imperial College London, London, UK

**Keywords:** Bilingualism, multilingualism, working memory, executive functions, cognitive development, bilingual advantage

## Abstract

Previous research has shown that cognitive development is sensitive to socio-economic status (SES) and multilinguistic experiences. However, these effects are difficult to disentangle and SES may modulate the effects of multilingualism. The present study used data from a large cohort of pupils who took part in the Study of Cognition, Adolescents and Mobile Phones (SCAMP) at ages 11–12 (T1) and 13–15 years old (T2). Cognitive measures were derived from tasks of cognitive flexibility, verbal, spatial and visuo-spatial working memory, speech processing and non-verbal reasoning. Using SES information collected through questionnaires (school type, level of deprivation, parental education and occupation), the sample was clustered into high/medium/low SES groups. Comparisons focused on 517 monolingual and 329 multilingual pupils in the high/low SES groups. Having controlled for multiple comparisons, the results indicated a significant beneficial effect of bilingualism in measures of working memory, visuo-spatial processing and non-verbal reasoning. These effects were present in both high and low SES individuals and sustained at both times of development, with a particularly significant improvement of working memory abilities in low SES bilinguals at T2 as compared to monolingual peers. Theoretical and practical implications of these findings are considered and guidance for educators is discussed.

## Introduction

Being able to communicate in more than one language is undoubtedly a remarkable skill and an obvious advantage in our modern multicultural societies. However, historically, the question of whether children who are born and raised in multilinguistic environments should learn two or more languages since the early stages of their lives has haunted parents and educators. These children are referred to in the literature as simultaneous or early multilinguals because they start acquiring their languages either since birth or at a very early stage of their life.

In cognitive psychology and psycholinguistics, multilingual and monolingual speakers have been extensively compared on measures of verbal performance, with tasks measuring linguistic skills, such as vocabulary acquisition (e.g. Oller, Pearson, and Cobo-Lewis 2007) or verbal fluency (Filippi, Ceccolini, and Bright 2021), and non-verbal performance, with a focus in particular on components of executive function (i.e. inhibition of irrelevant information, switching and shifting tasks and working memory processes – see Bialystok 2017, for an extensive review of the literature). These comparisons have generated conflicting results. Initially, monolinguals were seen to outperform bilinguals on a wide range of verbal and non-verbal measures (e.g. Saer 1923). These results, largely affected by poorly controlled socioeconomic status factors, inoculated the belief in the general public that children should be raised as monolinguals in avoidance of any possible unpleasant linguistic and cognitive delays. Still today, teachers in early years and primary schools in the UK and abroad, have concerns about multilanguage learning and it is not infrequent to hear stories in which parents are discouraged to raise their children as multilinguals (Festman, Poarch, and Dewaele 2017). More recent research has shown, on the contrary, that there is no real scientific ground to these concerns (e.g. Bialystok 2017; Filippi, D’Souza, and Bright 2019), even in atypical development (Dai et al. 2018; Drysdale, van der Meer, and Kagohara 2015; Filippi and Karmiloff-Smith 2013; Howard, Gibson, and Katsos 2021). In support of this claim, a recent report in the UK has shown that multilingual pupils have identical attainment to the national average at GCSE level, and they are more likely to achieve the English Baccalaureate than those with English as a first language (Hutchinson 2018).

The pioneering work of Bialystok and colleagues has shown consistently for at least three decades that bilingualism is not detrimental for cognitive development (Bialystok 2017). If anything, bilingual children have been often reported to outperform their monolingual peers in measures of executive function (EF), which includes the ability to inhibit irrelevant information, shift between tasks and update information in working memory (Miyake et al. 2000). This possible bilingual advantage has been observed in populations of different multicultural backgrounds and socio-economic statuses (e.g. Bialystok and Viswanathan 2009) and it has been explained, theoretically and practically, with the beneficial effect of constant language switching in daily life that in turn can enhance crucial attentional processes (Green and Abutalebi 2013; Green 1986, 1998; Bialystok 2017) that are construed in this case as domain general, and therefore able to influence skills outside of the language domain. Some authors have also provided tantalising evidence that lifelong use of multiple languages may translate in a protective effect on the ageing brain, with some studies reporting a potential delay of the onset of neurodegenerative disorders such as dementia and Alzheimer’s in multilingual speakers (e.g. Craik, Bialystok, and Freedman 2010; Woumans et al. 2015), although such studies suffer from a lack of random assignment to condition and are therefore open to confounds.

In recent years, the bilingual advantage has been questioned by some authors who have not been able to replicate the same findings (e.g. Morton & Harper, 2007; Paap and Greenberg 2013; Paap, Johnson, and Sawi 2015). The challenges of ensuring a ‘perfect’ match between monolingual and multilingual speakers, and the choice of sensitive tests that can rigorously measure even the more subtle individual differences, have made this line of study particularly difficult (see Filippi, D’Souza, and Bright 2019; Phelps and Filippi 2020 for an extensive review of the debate).

Morton and Harper were perhaps the first to highlight that the existing evidence for a possible bilingual advantage could have been affected by poorly controlled differences in participants’ ethnicity and socioeconomic status (SES). Between-group variability in SES has been consistently identified as an influential factor in studies of cognitive ability and school attainment in all populations, regardless of their linguistic backgrounds (e.g. Hackman et al. 2015; Lawson, Hook, and Farah 2018; Thomas 2017; Vrantsidis et al. 2020). Commonly, three key measures are used to capture SES in most studies: level and type of education, occupational status and household income (e.g. Filippi et al. 2020; 2021)

Large scale cross-sectional studies in which the monolingual and bilingual participants’ SES was rigorously controlled and homogeneous across the two groups have not provided any statistically significant evidence for a bilingual advantage in executive function (e.g. Filippi et al. 2020, 2021). The homogeneity in levels of SES (i.e. the lack of variability in the samples) could account for this null effect and possibly explain the inconsistent findings of the relationship between bilingualism and executive function.

Two recent studies seem to confirm this hypothesis. The first study by Naeem et al. (2018) examined the effects of bilingualism on executive function by comparing the performance of low and high SES young adult bilinguals and monolinguals on the Simon task, a task that has been employed in bilingual research extensively to measure components of executive function like inhibition, monitoring and updating (Miyake et al. 2000). Low-SES bilinguals outperformed low-SES monolinguals, that is, they responded faster when resolving the cognitive conflict presented in the Simon task. However, high-SES bilinguals and monolinguals did not differ (Naeem et al. 2018). These results raise the possibility that a multilingual experience may not be important in high-SES populations, but may help offset the negative impact of impoverished environments on cognitive development (Naeem et al. 2018; Turkheimer et al. 2003).

The second study by Grote, Scott, and Gilger (2021) provided further evidence for a possible bilingual advantage in executive function by comparing the performance of a group of Spanish-English bilinguals and two monolingual control groups (English and Spanish) of young children from low-SES backgrounds in California (USA). In three separate experiments targeting visual-spatial working memory, inhibitory and attentional control, they found that the bilingual children outperformed their monolingual peers in all measures. Both Naeem et al.’s (2018) and Grote et al.’s (2021) studies, although providing tantalising results, employed relatively small samples of a specific age (i.e. preschool children and young adults).

The current study aims to expand this understudied line of investigation and further explore how the contrast of different levels of SES (i.e. high vs. low SES) in combination with a multilinguistic experience can modulate executive function skills.

By contrasting only the high and low SES groups, this exaggerates the statistical effect of SES and therefore fulfils two aims: (1) it provides the strongest test of the hypothesis that bilingual executive function differences are actually a marker for SES-caused differences, and (2) it provides the conditions to replicate the previous observation that SES might moderate bilingual effects, whereby the cognitive differences occur in low SES groups but not high SES groups.

This study aimed to fill a gap in the literature in three key ways: (1) by examining a much larger sample than previous studies, (2) by specifically targeting a crucial age for the development of executive function skills, i.e. adolescence, (3) by following the participants’ cognitive development longitudinally from the age of 11 to the age of 15 years old.

## The SCAMP database and rationale for this study

We were offered the opportunity to follow up on Naeem et al.’s work (2018) by analysing and comparing the cognitive performance of multilingual and monolingual secondary school pupils who took part in the Study of Cognition, Adolescents and Mobile Phones (SCAMP) led by Imperial College, London, UK. SCAMP is a large cohort study in which over 8,000 pupils from 39 schools in London (UK) and surroundings were assessed with a wide range of cognitive measures at two different times of their development, one when attending Year 7 (11–12 years old) and the other when attending Year 9 (13–15 years old). From now on, these two phases of testing will be described as T1 and T2. Biographical, linguistic and socio-economic (SES) information was also collected through questionnaires (see Toledano et al. 2019, for more details).

These measures allowed us to identify the English monolingual and multilingual pupils from various linguistic backgrounds and select for analysis those in each linguistic group with the highest and the lowest SES scores in four dimensions, i.e. parental education, levels of deprivation and parental education and occupation status (Table 1).

**Table 1.** Sociodemographic data from the SCAMP dataset analysed in this study.

Questionnaires (socio-demographic)	Answers
English as a first language	0 = No 1 = Learned at the same time as another language 2 = Yes
Languages spoken within family	One or more choices among 23 different options
School type	0 = State, 1 = Independent
Carstairs postcode deprivation	1 = lowest, 5 = highest
Parental education	0 = did not attend university, 1 = attended university
Parental occupation	1 = lowest, 8 = highest

For parental occupation, the Office for National Statistics (ONS) classification was used. Parental occupation is a measure of each parent’s occupation status level. Participants were asked a series of standard questions about their mother’s and father’s occupations during the main assessment questionnaire. Studies have indicated that adolescent reporting of parental occupation level is reasonably accurate (Lien, Friestad, and Klepp 2001), and previous studies have used this method of assessing SES (Richter, Leppin, and Gabhainn 2006). Responses were coded as per the 8-class version of the NS-SEC (Rose and Pevalin 2003). Values were then re-coded such that a higher number indicates a higher SES category (Table 2). In the main assessment questionnaire, participants reported whether their mother attended university and whether their father attended university, which provided the parental education measures.

**Table 2.** Socio-economic status (SES) parental occupation coding categories.

SES level	Occupation description
8	Large employers and higher managerial and administrative occupations
7	Higher professional occupations
6	Lower managerial, administrative and professional occupations
5	Intermediate occupations
4	Small employers and own account workers
3	Lower supervisory and technical occupations
2	Semi-routine occupations
1	Routine occupations
–	Never worked or long-term unemployed

Carstairs postcode deprivation is an estimate of the deprivation level of the postcode area of the participant’s home address, relative to the area sampled by the SCAMP study. Home address was reported by the participants during the main assessment. A deprivation index score was estimated for each participant using the Carstairs index (Morgan and Baker 2006). Carstairs index values are calculated nationally, by taking weighted Z-score composite of four key economic indicators (Table 3) for each geographic area across England, using 2011 census data (Morris and Carstairs 1991). Scores were then normalised across the SCAMP study sample area, and individual values relative to these normalised scores were calculated for each participant’s reported home postcode. Data were then categorised into quintiles, with 1 = most deprived and 5 = least deprived, as mentioned in Table 1.

**Table 3.** Economic indicators used to calculate Carstairs postcode deprivation index measure.

Indicator	Description
Male unemployment	The proportion of economically active males seeking or waiting to start work
Lack of car ownership	The proportion of all persons in private households which do not own a car
Overcrowding	The proportion of all persons living in private households with a density of more than one person per room
Low social class	The proportion of all persons in private households with an economically active head of household in partly skilled or unskilled occupations, according to ONS-NSSEC classifications

The overarching question addressed in this work was whether the interaction between levels of SES and a multilinguistic experience can confer any significant effect in cognitive development – and whether any such effects target particular cognitive abilities. Specifically, we were interested in identifying which component(s) of executive function may be affected by this interaction, and in particular, whether we would replicate earlier findings of greater effects of multilingualism on cognition in lower SES groups. Another important aspect that we wanted to investigate was whether these effects were present in early adolescence (T1) and how they evolved through development of individuals followed longitudinally (T2).

## Methods

### Participants’ data selection

A total of 8119 individuals (39% males) were extracted from the SCAMP database. All participants completed a series of questionnaires that provided socio-demographic and linguistic information. Table 1 shows the data that were extracted for the purposes of this study.

The following filtering criteria for participants’ selection were applied: (1) Only individuals who took part to both T1 and T2 testing phases were selected; (2) The language categories were identified, based on the first language acquired and the language currently spoken with the family; (3) Only individuals with all the required SES scores were included. This resulted in a total number of 1447 individuals.

## Data clustering and statistical analysis

The filtered dataset was clustered by the four SES variables (normalised in *z*-scores) already shown in Table 1 (i.e. parental education, parental occupation, level of deprivation and school type). Cluster analysis returned three groups, high/medium/low SES. Following up the work of Naeem et al. (2018) we specifically focussed on the comparison between the high-SES and the low-SES participants (Table 4). Therefore, only the high and the low groups were selected for data analysis.

**Table 4.** Characteristics of the high and low SES groups identified in the cluster analysis results. The medium SES group was not included in further analyses.

Cluster	Tot.	Parental occupation	Level of deprivation	School type	Parental education	Monolinguals	Multilinguals
High SES	553	6.66 (1.06)	2.12 (1.18)	1.00 (0.00)	0.88 (0.32)	402	151
Low SES	306	4.63 (1.95)	3.93 (1.19)	0.00 (0.00)	0.00 (0.00)	116	190

The data clustering resulted in a total of 859 individuals (34% males) in high or low SES groups. Their mean age at T1 was 12.0 years old (SD = 0.4) and at T2 was 14.2 years old (SD = 0.5). Based on the linguistic experience data reported in the questionnaire, the individuals were subdivided in the following linguistic categories: (1) 517 English monolinguals; (2) 329 multilinguals (i.e. those who responded ‘1’ to the language question in Table 1).

The performance of multilingual and monolingual speakers in both SES groups was compared through a series of mixed analyses of variance (ANOVAs) examining each single variable at two points in development (T1 and T2). As noted in the *Cognitive Tasks* section below, the tests were administered in the same order and those presented at the beginning of the session had more participants than those presented towards the end of the experimental session. The number of participants in each single task at T1 and T2 is reported in Table 5.

**Table 5.** Cognitive tests from the SCAMP dataset examined in this study and the total number of participants with longitudinal data. The tasks were administered in a fixed order, as shown in the table.

Cognitive assessment	Test used	Monolinguals high/low-SES	Multilinguals high / low-SES
Cognitive flexibility	Trail Making Test (TMT)	398/111	150/183
Working memory	Backward Digit Span (BDS)	396/104	150/173
Spatial working memory	(SWM Errors and Strategy)	386/100	148/174
Speech processing	Speech-in-noise task (SPIN)	261/35	106/68
Non-verbal fluid intelligence	Cattell Culture Fair Test (CFT)	357/102	138/178
Visuospatial working memory	Corsi span task	184/29	87/61

### Targeted cognitive tests

Performance was analysed from six cognitive tests used in SCAMP to assess a broad range of executive functioning (EF), including cognitive flexibility (trail making test, TMT), working memory (backward digit span, BDS and spatial working memory task, SWM) and visuospatial working memory (Corsi span test). Measures of fluid intelligence (Culture fair task, CFT) and speech processing (speech in noise task, SPIN) were also included. These tests are generally used in bilingual research (e.g. Filippi et al. 2019; Filippi, D’Souza, and Bright 2019, Filippi et al. 2020; Filippi, Ceccolini, and Bright 2021; Seçer 2016) to test theoretical frameworks, such as the Inhibitory Control Model (Green 1986, 1998). The model predicts that a domain-general inhibitory control mechanism is required in order to resolve the competition from both languages, suppress the language not in use and select the target one. Green’s putative inhibitory control mechanism is frequently the proposed locus of transfer effects from the control of the bilingual’s languages to possible beneficial effects in wider cognitive skills. Hence cognitive control skills are a common target for the exploration of the beneficial effects of multilingual experiences.

The tests were computerised and administered in a fixed order (see Table 3) in the classroom, in order to maximise the participant numbers for the most important measures in the overall SCAMP study.

A brief description of each test and the testing procedure is provided below.

#### Trail making test (TMT)

This task was a computerised version of the TMT used in neuropsychological studies (Tombaugh 2004). It was performed first as it provides measures of processing speed, visuospatial processing, motor sequencing and a well-validated measure of EF skills, namely cognitive flexibility and switching. Three conditions were completed in a fixed order: (i) the Dot condition, (ii) the Letters condition and (iii) the Letters and Numbers switching condition. Each condition included 20 dots. Cognitive flexibility, or switching ability, was measured by calculating the unstandardised residuals regressing response time in the Letters condition from response time in the Letters/Numbers condition. Higher values reflect greater switching cost, i.e. poorer cognitive flexibility.

#### Backward digit span task (BDS)

This test was performed second as it is a commonly used verbal WM test, which shows performance improvement during late childhood and adolescence, significant individual differences, and is a predictor of academic performance. In the standard procedure, a sequence of numbers is read aloud to the participant, who then repeats the sequence back in reverse order (e.g. Dumontheil and Klingberg 2012**)**. In this version, single digit numbers were displayed on the computer screen, one-by-one. At the end of each sequence, participants were asked to reproduce the sequence of numbers in the reverse order by clicking on a numerical response pad. A staircase procedure (Levitt procedure) was used to reduce the time taken on the main phase of the task. The key measure was the average of mean level passed and mean level failed, reflecting verbal WM capacity (see Maes, Pirani, and Booth 2021 for more published information about this task).

#### Spatial working memory (SWM) task

This task was adapted from the Spatial Working Memory task of the CANTAB battery **(**Luciana and Nelson 2002). It was performed third as it is a test of both visuospatial WM and a higher order aspect of EF related to strategy. In this task, participants are required to visit a sequence of locations marked by ringing phones. They are instructed to ignore any locations that they have previously visited. There were four levels, completed once in ascending order, with 4, 6, 8, 10 items. Within search errors (WSE) on the task are when the participant returns to a location that has been previously searched on the same trial. Between search errors (BSE) are returning to a location that has been the active location (the ringing phone) in a previous trial in the same block. There were two key measures: (i) The total number of errors (WSE and/or BSE) made throughout the task, reflecting spatial WM capacity and (ii) the strategy score reflecting planning/strategy use, which was determined by checking whether participants consistently started their search at the same location.

#### Speech in noise (SPiN) task

The Speech in Noise task targeted speech processing. It was selected because, although quite perceptual in nature, performance is also linked to higher level language abilities, and the task is sensitive to individual differences in this age range. In this version, an audio stimulus was presented at a specified signal-to-noise ratio (SNR) saying, for example ‘show the dog where the *blue six* is’. Participants were instructed to click on a number to respond. The SNR varied according to a Levitt staircase procedure to identify the SNR level at which participants had 50% accuracy. More negative scores indicated better performance, i.e. that participants could identify the instructions with higher levels of noise.

#### Cattell’s culture fair task (CFT)

This is a computerised version of a standardised visuospatial reasoning test, assessing non-verbal reasoning (Cattell and Cattell 1949). It was included in the SCAMP battery as a marker of interest for teachers and parents and predicts academic performance. Scores may be affected by the fact that certain primary schools in the UK train their pupils on this type of test. Two subtests were used: the Odd One Out task and the Complete the Pattern task; stimuli were taken from Cattell's Culture Fair Test Form A Scale 2. Each subtask had a three-minute time limit, and participants completed as many items as possible in that time (maximum 14 items in Odd One Out, max 11 in Complete The Pattern). The key measure was the total number of correct trials on the two subtasks, which reflected non-verbal reasoning and served as a proxy for fluid intelligence/non-verbal IQ.

#### Corsi task

A computerised version of the Corsi task (Corsi 1972) was used, similar to that used in the Automated Working Memory Assessment (AWMA – Alloway 2007) and by Dumontheil and Klingberg (2012). This task targets visuospatial WM but does not have a strategic component. The test was included towards the end of the battery and it has been used to predict maths performance and served as an additional visuospatial WM measure. Circles lit up one-by-one in a fixed pseudo-random sequence in a 4 × 4 grid and participants are asked to remember and reproduce the sequence. The task followed a similar staircase procedure to the BDS task. As in the BDS, the key measure was the average of the mean level participants passed and the mean level they failed.

## Results

The statistical analyses were carried out following the same task order reported in Table 2 above. Descriptive statistics are shown in Table 6.

**Table 6.** Means and standard deviations (in brackets) of cognitive measures in of monolingual and multilingual children with high vs. low SES and at two points in their development (T1 – age 11–12 and T2 – age 13–15 years old).

Test	Time	Monolinguals		Multilinguals	
		High SES	Low SES	High SES	Low SES
Trail Making Test (TMT)^a^	T1	1.75 (.53)	1.79 (.57)	1.67 (.40)	1.92 (.66)
	T2	1.71 (.50)	1.79 (.57)	1.67 (.45)	1.83 (.58)
Backward Digit Span (BDS)	T1	4.47 (.86)	3.90 (.73)	4.68 (.88)	4.00 (.89)
	T2	4.80 (.94)	4.09 (.92)	4.89 (.80)	4.40 (.95)
Spatial Working Memory – Errors (SWM)^a^	T1	24.10 (12.0)	29.33 (12.0)	22.67 (10.5)	30.22 (12.9)
	T2	19.96 (10.8)	26.91 (12.0)	19.25 (10.1)	23.17 (12.2)
Spatial Working Memory – Strategy (SWM)^a^	T1	11.40 (4.8)	10.80 (2.9)	11.45 (4.7)	10.83 (2.9)
	T2	9.37 (3.1)	10.91 (2.5)	9.20 (2.7)	10.13 (2.9)
Speech-in-noise task (SPIN)^a^	T1	−6.6 (2.8)	−6.9 (2.6)	−6.6 (2.6)	−5.8 (3.3)
	T2	−7.1 (3.0)	−6.5 (2.2)	−6.9 (3.0)	−5.6 (3.6)
Cattell Culture Fair Test (CFT)	T1	14.35 (3.0)	13.08 (3.4)	15.34 (3.0)	13.20 (3.5)
	T2	16.0 (3.0)	13.92 (3.4)	16.54 (3.2)	15.11 (3.6)
Corsi span task (CORSI)	T1	5.20 (.71)	4.72 (.73)	5.34 (.77)	5.04 (.70)
	T2	5.64 (.80)	4.94 (.97)	5.64 (.79)	5.37 (.89)

^a^ Lower TMT, SWM and SPiN scores indicate better performance.

### Cognitive flexibility (TMT)

A 2 × 2 × 2 mixed ANOVA with *time* (T1 and T2) as the within-subjects factor and *language group* (multilinguals, monolinguals) and *SES* (high, low) as the between-subjects factors, revealed a significant main effect of SES, *F*(1,838) = 17.9, *p *< .001, $\eta_{p}^{2}$^ ^= .02, and a significant interaction between language groups and SES, *F*(1,838) = 4. 5, *p = *.034, $\eta_{p}^{2}$^ ^= .005. Post-hoc *t*-tests did not survive a Bonferroni adjusted alpha level of .0125 per comparison (.05/4)

**Figure 1.**
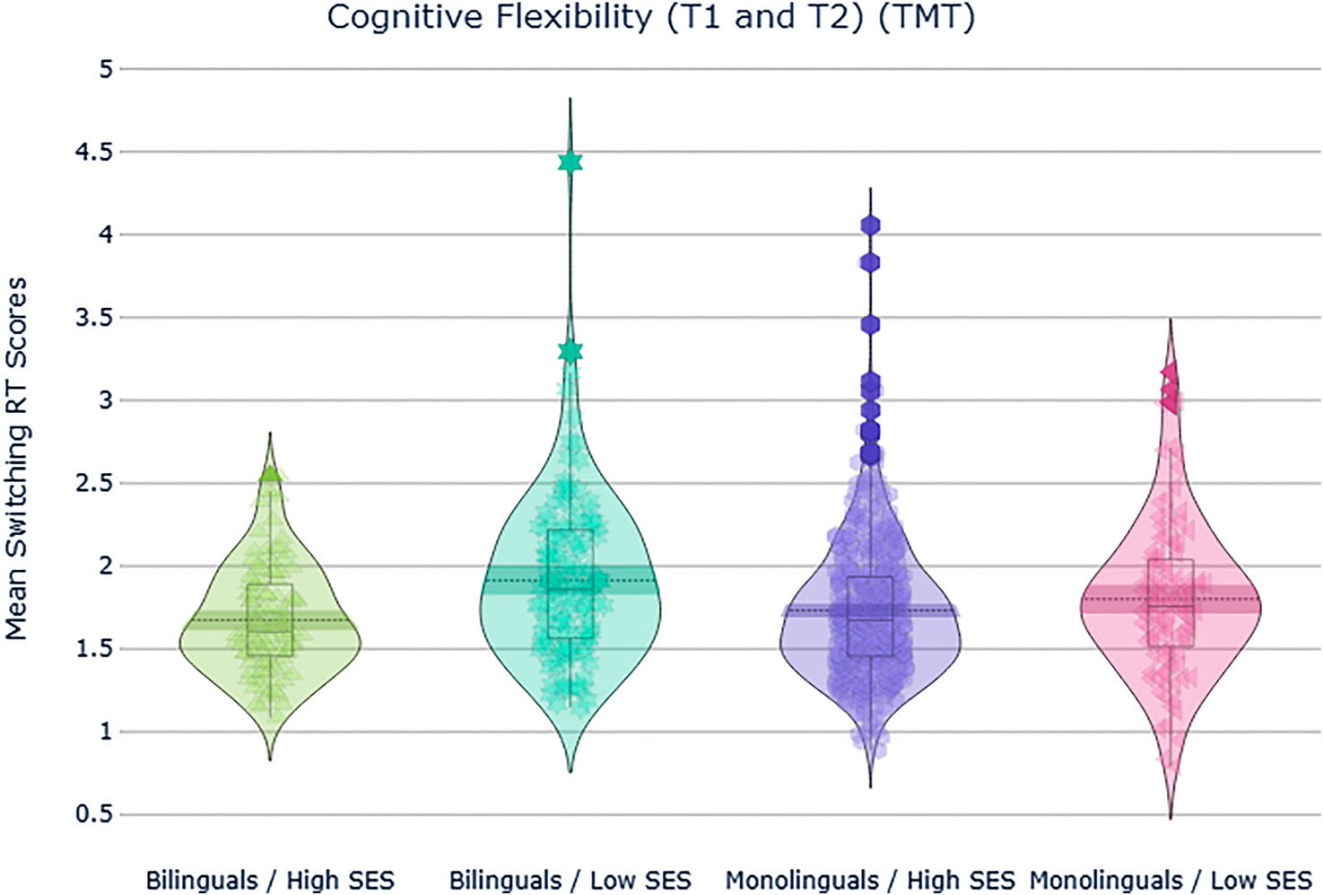
Interaction between language group and SES observed for the cognitive flexibility (switching) Trail Making across T1 and T2. The response time switching cost measure is the unstandardised residuals obtained from regressing response time in the Letters condition from response time in the Letters/Numbers (switching) condition. Higher values reflect greater switching cost, i.e. poorer cognitive flexibility.

High-SES participants had comparable performance regardless of their linguistic experience at both T1 and T2 (Figure 1). All the other main effects (i.e. time, language group) and interactions (i.e. time*language group, time*SES, language group* SES and time*language group*SES) were non-significant (*p *> .05).

In summary, high SES adolescents outperformed low SES peers at both timepoints in development on a measure of cognitive flexibility, regardless of their linguistic experience. A significant interaction between language groups and SES was observed. It was driven by relatively greater switching costs in low SES bilinguals then in low SES monolinguals. However post-hoc *t*-tests were not significant at a Bonferroni adjusted alpha level of .0125.

#### Verbal working memory (BDS)

**Figure 2.**
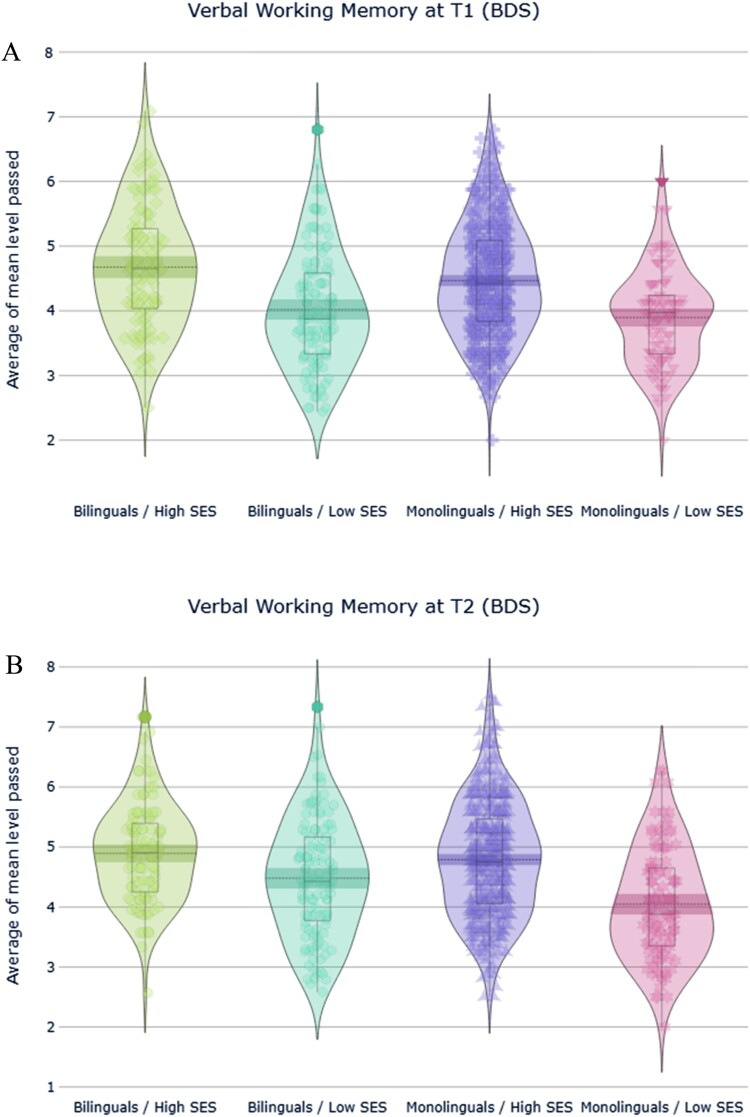
Results of verbal working memory capacity at T1 (a) and T2 (b). The Backward Digit Span score was calculated as the average of the mean working memory load level passed and mean level failed.

ANOVA revealed significant main effects of SES with a medium effect size, *F*(1,819) = 102.8, *p *< .001, $\eta_{p}^{2}$^ ^= .11, and of language group, *F*(1,819) = 8.6, *p *= .003, $\eta_{p}^{2}$^ ^= .010; and time, *F*(1,819) =  63.2, *p *< .001, $\eta_{p}^{2}$^ ^= .07, with small effect sizes, indicating that levels of SES and language experience both modulated verbal working memory performance in adolescence. The two-way interactions between SES and language group, language group and time and SES and time were all non-significant (*p *> .05). However, the three-way interaction between SES, language group and time was significant, *F*(1,819) = .5.2, *p *= .02, $\eta_{p}^{2}$^ ^= .006. Bonferroni corrected post-hoc analysis showed a reliable best performance in high SES bilinguals at T1 (*p *= .01) and low SES bilinguals at T2 (*p *= .004) both with large effect size, (Cohen’s d = .87 and .93 respectively). These results show, overall, that the participants with higher SES performed better than lower SES. However, bilinguals showed a general better performance than monolinguals, in the high SES at T1 and in the low SES group at T2 (Figure 2a and b).

#### Spatial working memory (SWM)

**Figure 3.**
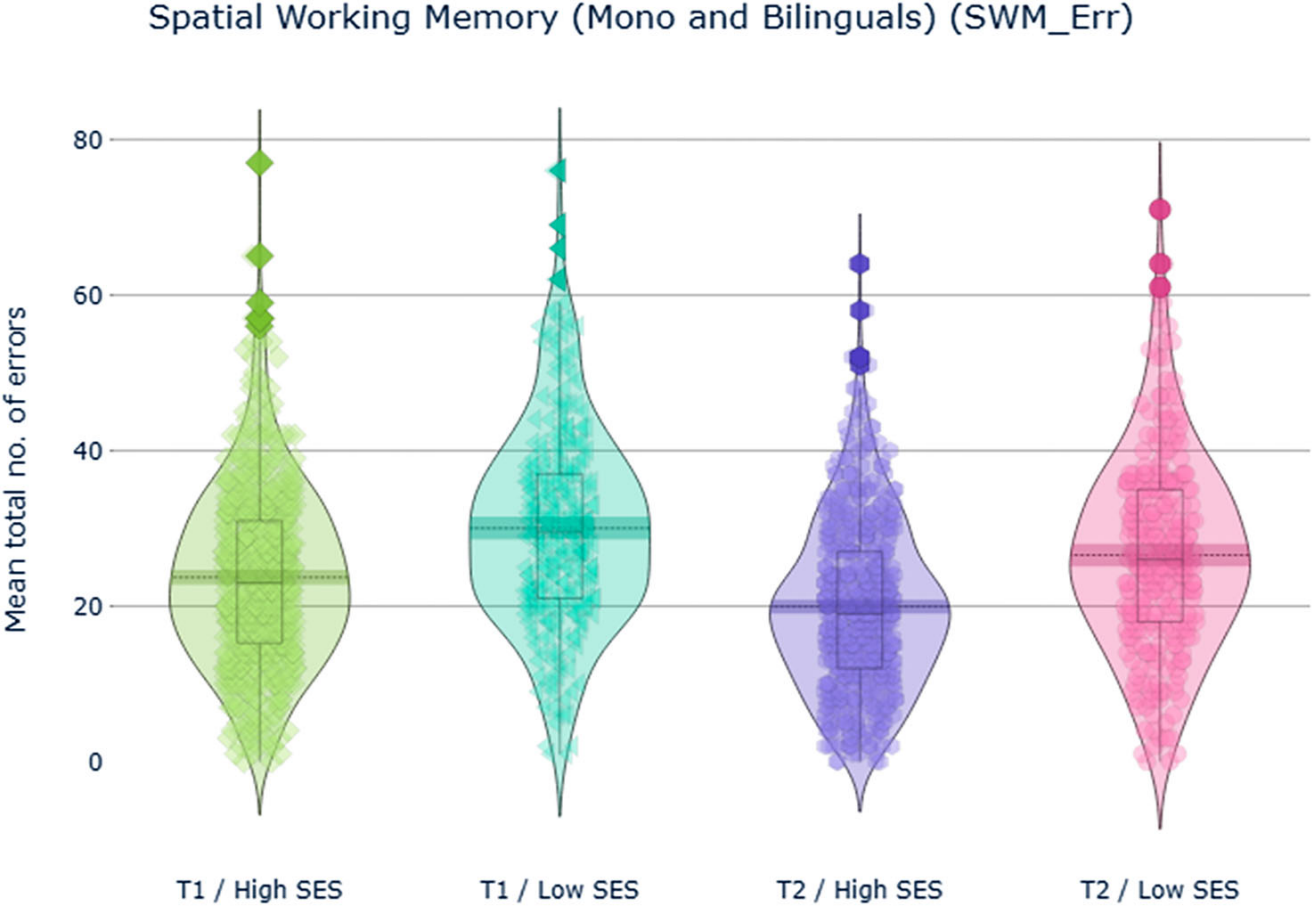
Participants’ performance in the Spatial Working Memory task: total number of errors (within-search and/or between-search) made throughout the task, measuring spatial working memory capacity at T1 (a) and T2 (b).

First, ANOVA for total number of errors was performed. As illustrated in Figure 3, the analysis revealed a highly significant main effect of SES with a medium effect size, *F*(1,804) = 83.8, *p *< .001, $\eta_{p}^{2}$^ ^= .09 and time, *F*(1,804) = 36.8, *p *< .001, $\eta_{p}^{2}$^ ^= .05, but there was no significant effect of language group nor reliable interactions between language group, SES and time (*p *> .05).

**Figure 4.**
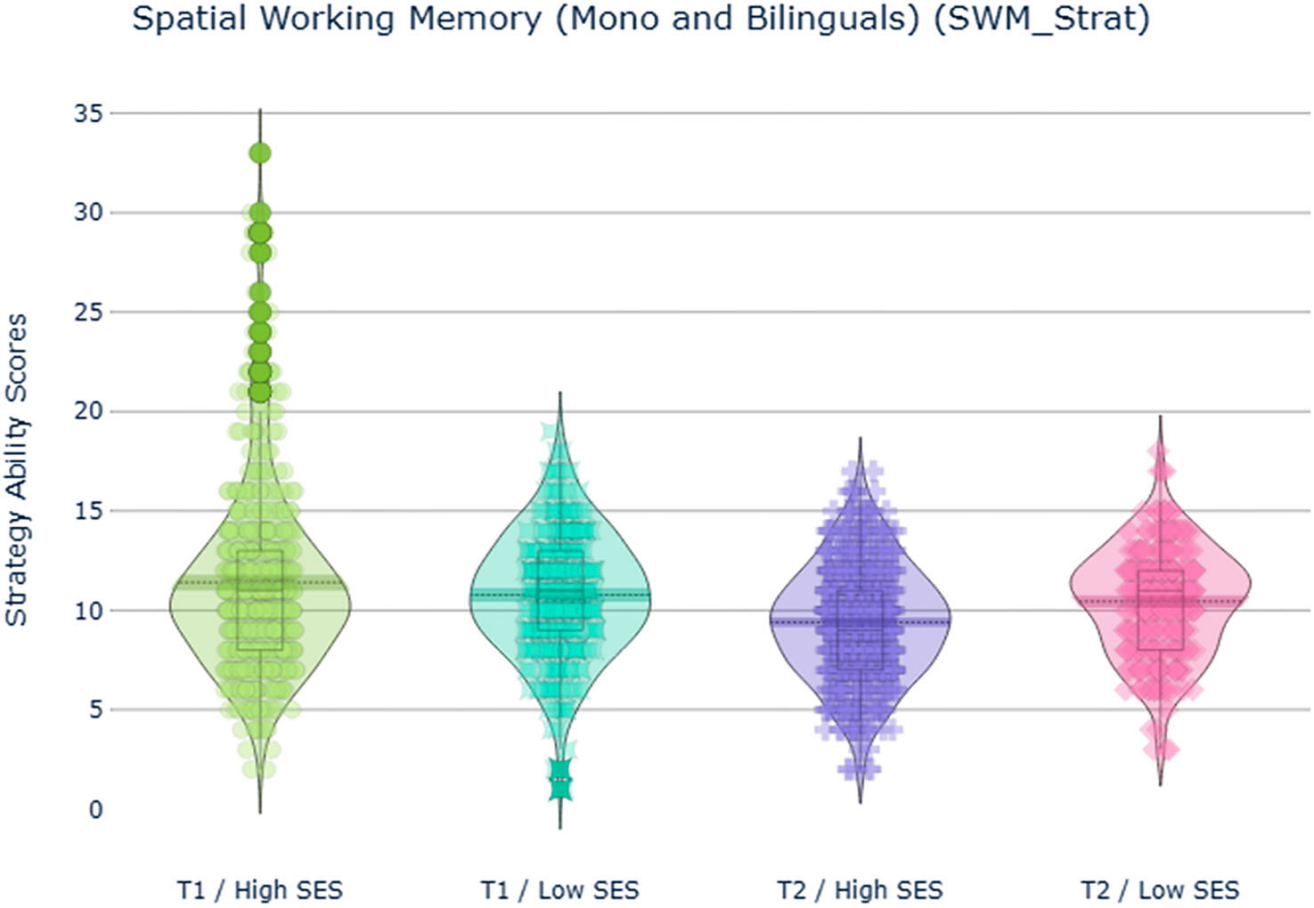
Strategy scores in the Spatial Working Memory task, reflecting planning/strategy use, which was determined by checking whether participants consistently started their search at the same location. Comparison between high and low SES participants at T1 (a) and T2 (b).

The second ANOVA was performed for strategy scores. There were non-significant main effects of language group and SES (*p *> .05), but there was a significant main effect of time with a small effect size, *F*(1,804) = 40.7, *p *< .001, $\eta_{p}^{2}$^ ^= .05, which was modulated by a significant interaction between SES and time, *F*(1,804) = 25.0, *p *< .001, $\eta_{p}^{2}$^ ^= .03. Bonferroni corrected *t*-tests indicated that the performance of high SES individuals significantly improved between T1 and T2 (*p *< .001), while the low SES group show no difference in performance between timepoints (*p *> .05) (Figure 4a and b). All the other main effects and interactions were non-significant (*p *> .05).

Taken together, these results indicated that levels of SES play a significant role for accuracy, a result that was confirmed also for the strategy performance, which reflected participants’ planning in the task. Participants made fewer errors at T2 (i.e. their performance improved through development). For strategy only high SES adolescents showed an improvement in performance over time.

#### Speech processing (SPIN)

All results of this analysis were not significant (*p *> .05). Both linguistic groups had a comparable performance regardless of their SES and time of testing.

### Cattell culture fair test (CFT)

ANOVA for total number of correct trials was performed. The analysis revealed significant medium size main effects of SES, *F*(1,771) = 80.6, *p *< .001, $\eta_{p}^{2}$^ ^= .095, and time, *F*(1,771) = 77.3, *p *< .001, $\eta_{p}^{2}$^ ^= .09, and a small size main effect of language group, *F*(1,771) = 4.0, *p *= .046, $\eta_{p}^{2}$^ ^= .005. All interactions were non-significant (*p *> .05).

**Figure 5.**
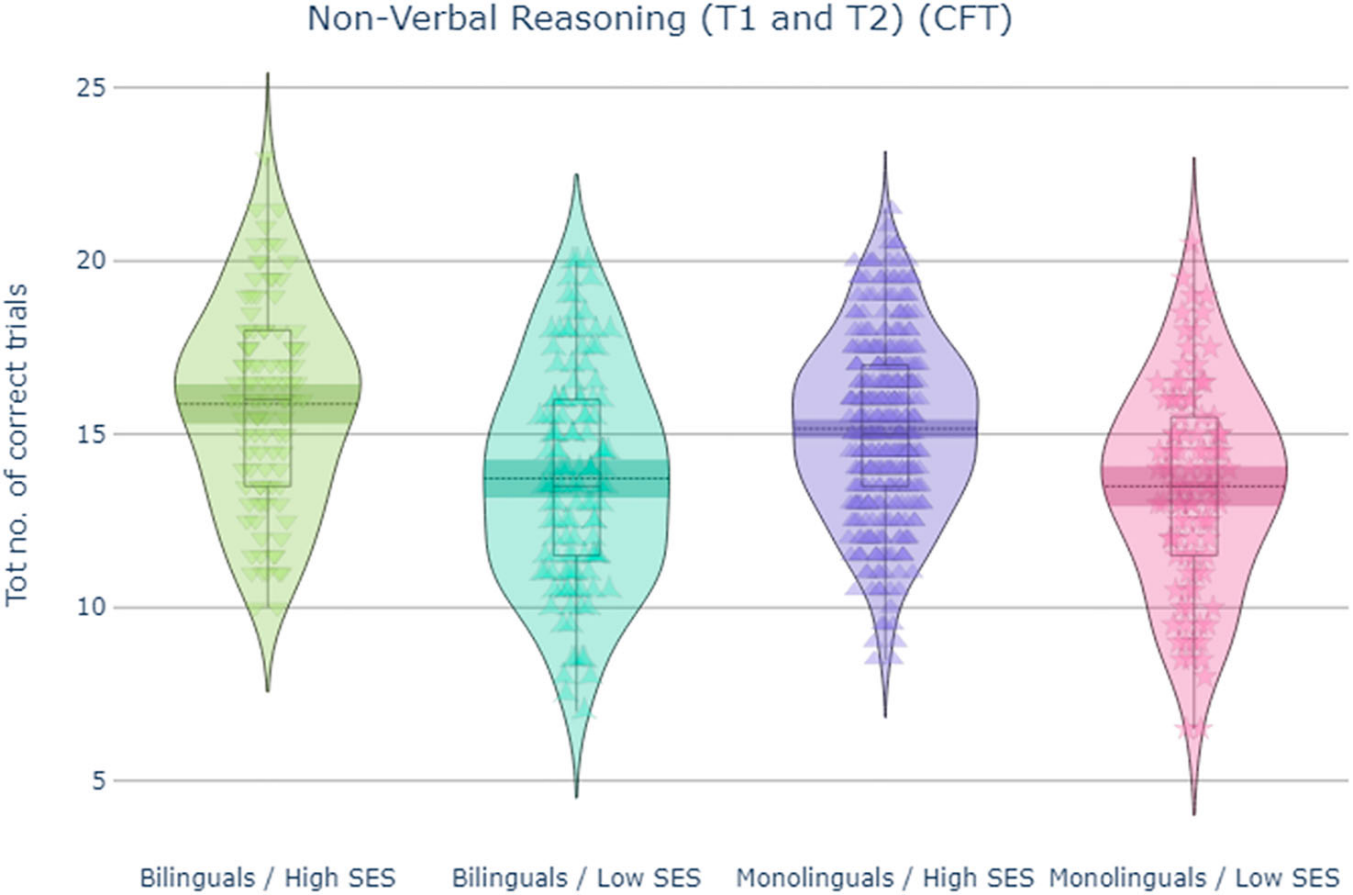
Total number of correct trials on the two Cattell’s Culture Fair subtasks across T1 and T2, which reflected non-verbal reasoning and served as a proxy for fluid intelligence/non-verbal IQ.

Overall, analysis of fluid intelligence results, illustrated in Figure 5, indicated that socio-economic status and language groups independently affected scores, with better performance observed in high than low SES groups and in bilinguals compared to monolinguals, across the two timepoints.

#### Corsi span task (CORSI)

**Figure 6.**
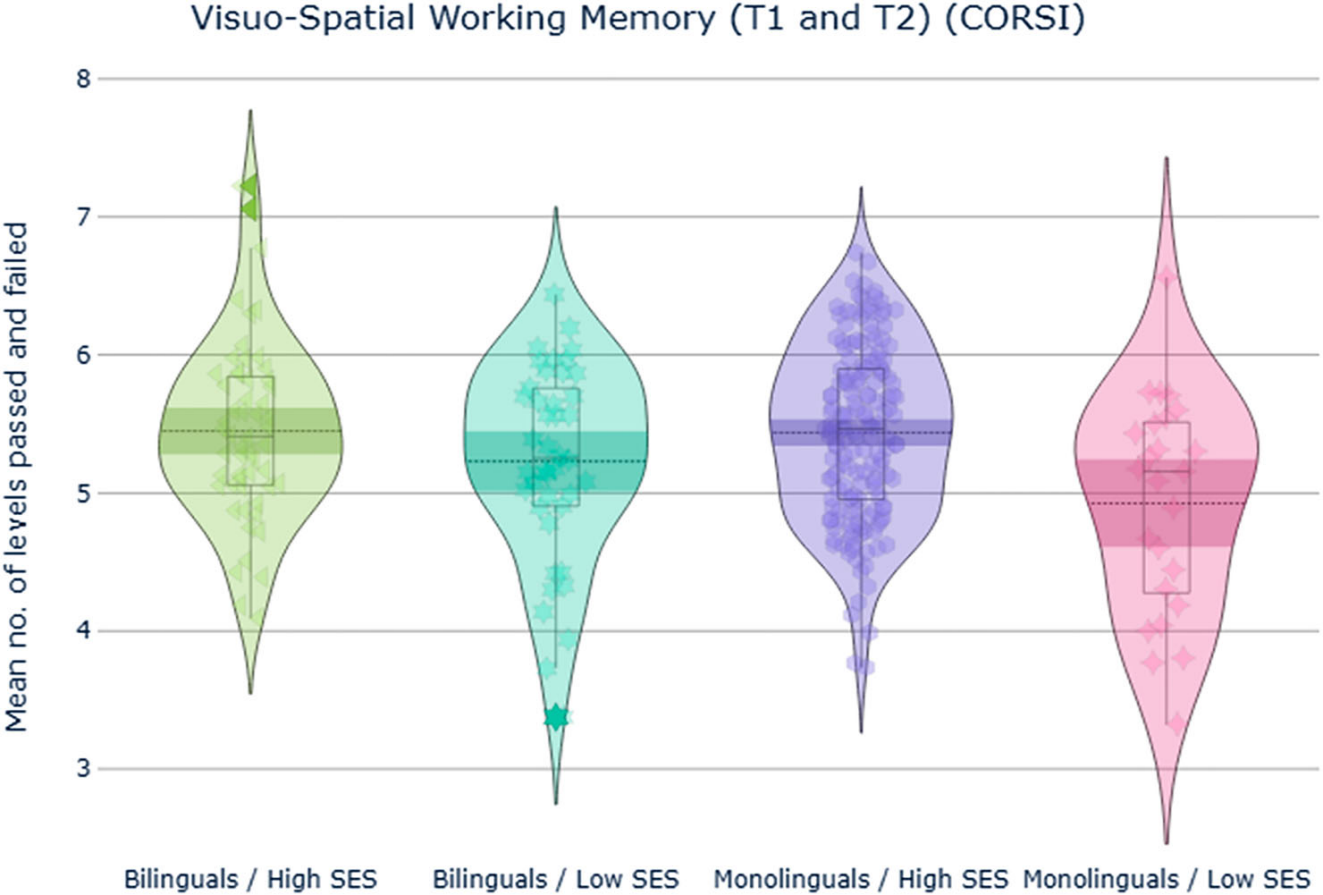
Participants’ performance in the Corsi task across T1 and T2, measured by averaging the mean level participants passed and the mean level they failed.

ANOVA revealed a highly significant main effect of SES, *F*(1,357) = 25.8, *p *< .001, $\eta_{p}^{2}$^ ^= .07, language group, *F*(1,357) = 6.5, *p *= .011, $\eta_{p}^{2}$^ ^= .02, and time *F*(1,357) = 35.1, *p *< .001, $\eta_{p}^{2}$^ ^= .09 (Figure 6). While performance on the task improved between the two time points, adolescents from higher SES showed better visuospatial working memory performance than adolescents from lower SES, and bilingual children showed a better performance than monolingual peers across development. All interactions were non-significant (*p *> .05).

## Summary of key results

In sum, main effects of language group were found for working memory (BDS) non-verbal reasoning (CFT) and spatial working memory (CORSI), with the average $\eta_{p}^{2}$ of .012. Main effects of SES were found for all tasks (average $\eta_{p}^{2}$ of .074), with the exception of the spatial working memory strategy condition and speech processing (SPIN). Interactions of language group and SES were only found for the cognitive flexibility task (TMT) with a $\eta_{p}^{2}$ of .007. However, the interaction did not survive post hoc tests corrected for multiple comparisons. There was a 3-way interaction between language group, SES and time in the working memory task (BDS) with the $\eta_{p}^{2}$ of .007.

## Discussion

This study examined the cognitive performance of a large cohort of children at two different stages of their development, at age 11–12 (T1) and 13–15 (T2) years old. They were tested as part of a wider research project targeting the effects of mobile phones use on cognitive development, the SCAMP project. A subset of 859 participants was selected from a larger database of 8119, following specific criteria to identify those who had longitudinal data, who were multilinguals and monolinguals, and who were clustered in high vs, low socio-economic groups. Their performance on a battery of six tasks measuring cognitive flexibility, verbal, spatial and visuo-spatial working memory, speech processing and non-verbal reasoning, was compared.

Performance on most tasks except the Trail Making task, measuring cognitive flexibility and the SPiN, measuring speech processing, improved with age during early adolescence. In addition, adolescents in the higher SES group outperformed adolescents in the lower SES group on all tasks except the SPiN, and the strategy measure of the spatial working memory task. An interaction between SES group and time indicated that low SES participants performed better than high SES participants on the SWM strategy measure, but only at T2. A general bilingual advantage was found in tasks measuring verbal working memory (BDS), non-verbal reasoning (Cattell’s culture fair) and visuo-spatial working memory (Corsi span task), across timepoints. Interactions between SES and language group were observed on two measures. There was a cross-over SES by language group interaction on the TMT measure, with a bilingual advantage for cognitive flexibility in the low SES but not the high SES group. In the BDS verbal working memory measure there was a relatively greater bilingual advantage for high SES adolescents at T1 and low SES adolescents at T2. The results suggest that if there is a bilingual advantage in verbal working memory, this is not sustained across adolescence in high SES individuals and may emerge during early adolescence in low SES individuals.

The findings of this study support a body of evidence that levels of SES have a significant impact on executive functions. Overall, they confirmed a general positive effect of high SES on cognitive development regardless of the participants’ linguistic experience, a finding that has been widely reported in the literature (e.g. Hackman et al. 2015; Lawson, Hook, and Farah 2018; Vrantsidis et al. 2020). However, they also indicated some significant results in favour of a beneficial and sustained effect of multilingual acquisition. Both high and low SES multilingual participants demonstrated a significant and consistent advantage over monolinguals in a visuo-spatial task across development. This result corroborates previous findings in which bilingual young adults outperformed monolinguals in tasks measuring visuo-spatial abilities (Kerrigan et al. 2017).

The only measure in which levels of SES modulated differences between the two linguistic groups was in the domain of verbal working memory. Here, we observed a bilingual advantage of high SES bilinguals at T1 but not at T2 and, on the contrary, a low SES bilingual advantage at T2 but not at T1. Importantly, this was in the context of a main effect of SES whereby higher SES adolescents outperformed lower SES adolescents across time points. If genuine, this pattern would indicate that multilingualism enriches working memory processes at an early age in high SES bilinguals, whereas possible beneficial effects of multilanguage acquisition may come to the fore later in development in low SES bilinguals. This result is particularly intriguing because differences in working memory processing between bilingual and monolingual children matched by SES are not usually found, (e.g. Filippi et al. 2015, 2020; Filippi, Ceccolini, and Bright 2021). However, as mentioned in the introduction, these studies did not contrast different levels of SES. Speculatively, these positive effects in working memory processes may be the basis of superior performance in executive function tasks observed at a later age in low SES individuals, as reported in Naeem et al. (2018).

In contrast, only main effects, i.e. sustained effects, of language group were found on two measures using visuospatial stimuli, the visuospatial working memory (Corsi) and visuospatial reasoning (Cattell Culture Fair) measures. SES has been associated with differential exposure to reading and speech (e.g. Chondrogianni and Marinis 2011), which may be why interactions with SES were observed with the verbal measures, but not the visuospatial measures, which may account for this differential pattern.

Another important consideration to be made concerns the specific executive function components that may be affected by a multilinguistic experience, in particular how bilinguals use their languages in everyday life. Why, for example, bilingualism may be beneficial in some cognitive processes but not in others? The present study does not offer a definite answer to this question.

The results showed that bilingualism may offer some positive effects in tasks measuring visuo-spatial abilities and working memory. The literature often reports positive effects of bilingualism in several domains of executive function, and across the lifespan. These include inhibitory control, selective attention, cognitive flexibility (e.g. switching) and updating (see Bialystok 2017 for a review).

However, inconsistent findings reported in the literature may be related to the specific individual linguistic skills and environmental experiences of the participants and, more importantly, did not specifically focus on low-SES populations.

Theories of multilanguage acquisition try to explain how managing and using two or more languages in a single mind affects cognitive processes. However, none of them addresses socio-cultural factors that might be at the basis of the possible effects of bilingualism on brain development. One of the most recent and influential theoretical frameworks, the Adaptive Control Hypothesis (ACH – Green and Abutalebi 2013) proposes that language control processes, in particular speech production, adapt to the cognitive demands. The main assumption is that different interactional contexts may pose different demands on these processes in bilingual speakers. The interactional contexts refer to how bilingual speakers use their languages in everyday life. For example, multilinguals can use their languages separately (e.g. L1 at work and L2 at home). In other cases, both languages can be used in a mixed modality. This implies frequent switching between languages that may occur within a conversation or even within the same sentence. The ACH makes specific predictions about the relationships between different linguistic experiences and executive processing, such as selective attention, goal maintenance, conflict monitoring and planning. New experimental work with a sample of bilingual Mandarin/English speakers seems to support these predictions: participants who switch between their languages more often had a better performance in executive function tasks measuring inhibitory control (Han, Li, and Filippi 2021). Speculatively, it may be possible the cognitive advantages are modulated by different levels of SES in which specific linguistic habits are present. For example, studies that compared primary school children who can read and write in two languages (i.e. biliterate bilinguals) to those who cannot (i.e. monoliterate bilinguals) have shown that a deeper knowledge of languages is associated with enhanced development of cognitive abilities (Dosi et al. 2016), and grammar and word reading skills (Leider et al. 2013). It may also be possible that some communities use their languages more often than others, for example, by offering more opportunities for children to engage in extra-curricular learning to preserve cultural heritage. On this point, it would be interesting for future research to investigate on specific linguistic habits in minority groups and communities of different ethnicity.

Indeed, the results of this study, as well as Naeem et al. (2018) and Grote, Scott, and Gilger (2021), suggest that a more comprehensive theoretical framework should include assumptions on the relationship between linguistic background, levels of SES and executive function. Unfortunately, in the current study, we did not have the opportunity to analyse the participants’ linguistic experience in great detail and some caveats should be acknowledged.

The SCAMP project did not specifically aim to collect linguistic information that is usually acquired in bilingual research, like the total number of languages known by each participant, the levels of proficiency and literacy in those languages, the daily use of each language (e.g. whether participants ‘switched’ between languages frequently in their habitual communication with others). This information, if available, would have given us the opportunity to explore the data more in detail and possibly identify some specific patterns of multilingualism that could be more strongly associated with cognitive development.

Nevertheless, some important strengths of this study should also be acknowledged. First, information about the participants’ socio-economic status allowed us to conduct a cluster analysis in which we were able to clearly separate groups with higher and lower SES. Second, we conducted our analysis on a large number of participants, which addresses one of the most criticised factors in the bilingual literature regarding statistical reliability. Third, we had the opportunity to examine the participants’ performance at two different times in their development and determine whether there were some baseline effects conferred by multilanguage acquisition and whether these effects sustained across development.

## General conclusions

Overall, this study has provided further evidence that early multilanguage acquisition cannot be considered a matter of concern for cognitive development. If anything, longitudinal analysis on the SCAMP dataset has shown that there might be a positive developmental effect of multilingualism, including within the more disadvantaged multilingual pupils, with a particular focus on working memory processing.

This study warrants further avenues of research specifically targeting the disadvantaged population and extending the investigation to qualitative analyses of linguistic exposure (e.g. use of languages at home, in the school, with friends and extended family), a variety of verbal and nonverbal measures and a convergence of methodologies, including neuroimaging techniques.

A final remark: The study of multilingualism has been beset with controversy, generated by the dichotomy between *advantages* or *disadvantages* for cognitive development. However, the possible benefits of a multilingual upbringing go well beyond mere cognitive aspects. In the UK, there are more than 1 million pupils who are learning English as an additional language (EAL) and they are not a homogeneous group.

It is therefore crucial for scientists in this field to engage with educators and policy makers and work together to dissipate any misleading information and help the pupils achieve their full potential while preserving their cultural heritage.
